# A Comprehensive Review of Our Current Understanding of Red Blood Cell (RBC) Glycoproteins

**DOI:** 10.3390/membranes7040056

**Published:** 2017-09-29

**Authors:** Takahiko Aoki

**Affiliations:** Laboratory of Quality in Marine Products, Graduate School of Bioresources, Mie University, 1577 Kurima Machiya-cho, Mie, Tsu 514-8507, Japan; aoki@bio.mie-u.ac.jp; Tel.: +81-59-231-9569; Fax: +81-59-231-9557

**Keywords:** red blood cell, glycoproteins, biological function, glycophorin, band 3, O-linked oligosaccharides, *N*-acetylneuraminic acid, *N*-glycolylneuraminic acid

## Abstract

Human red blood cells (RBC), which are the cells most commonly used in the study of biological membranes, have some glycoproteins in their cell membrane. These membrane proteins are band 3 and glycophorins A–D, and some substoichiometric glycoproteins (e.g., CD44, CD47, Lu, Kell, Duffy). The oligosaccharide that band 3 contains has one N-linked oligosaccharide, and glycophorins possess mostly O-linked oligosaccharides. The end of the O-linked oligosaccharide is linked to sialic acid. In humans, this sialic acid is *N*-acetylneuraminic acid (NeuAc). Another sialic acid, *N*-glycolylneuraminic acid (NeuGc) is present in red blood cells of non-human origin. While the biological function of band 3 is well known as an anion exchanger, it has been suggested that the oligosaccharide of band 3 does not affect the anion transport function. Although band 3 has been studied in detail, the physiological functions of glycophorins remain unclear. This review mainly describes the sialo-oligosaccharide structures of band 3 and glycophorins, followed by a discussion of the physiological functions that have been reported in the literature to date. Moreover, other glycoproteins in red blood cell membranes of non-human origin are described, and the physiological function of glycophorin in carp red blood cell membranes is discussed with respect to its bacteriostatic activity.

## 1. Introduction

The blood of mammals, such as humans, as well as of birds, reptiles, and teleosts, contains red blood cells (erythrocytes). Human red blood cells, which are the most commonly studied by researchers, are approximately 7 μm in diameter, and their centre has a dented discoid shape.

**Figure 1:**
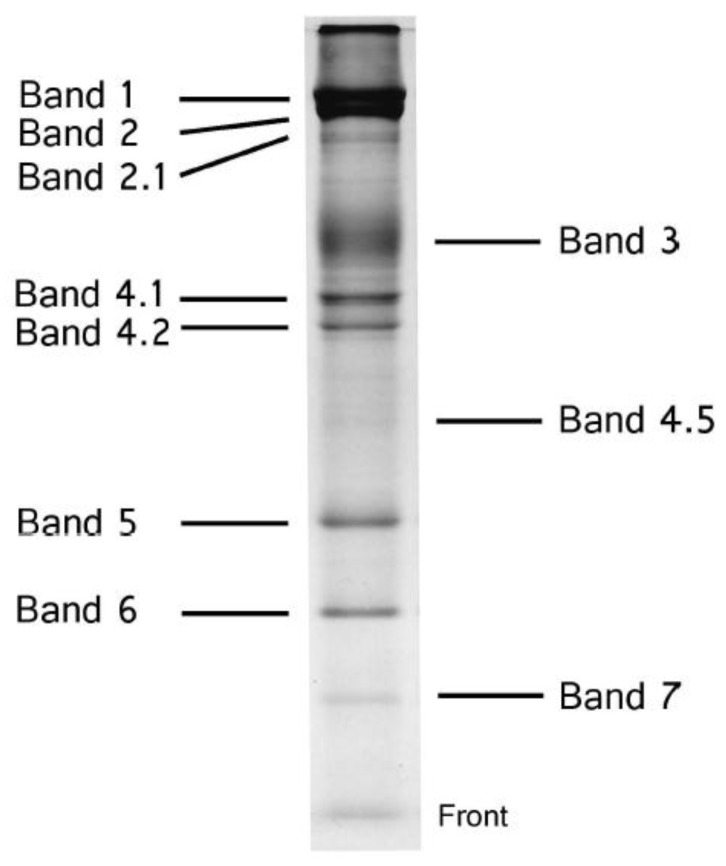
Sodium dodecyl sulfate (SDS)-polyacrylamide gel electrophoresis of human red blood cell (RBC) membranes. Healthy human RBC membranes stained with Coomassie brilliant blue. Electrophoresis was performed according to the method of Laemmli [2].

Studies on biological membranes normally use human red blood cells (RBC) because these erythrocytes have no nuclei and are easy to obtain for cell membrane preparation. For the preparation of membrane proteins, it is necessary to solubilize the phospholipid bilayer by using detergents. Fairbanks et al. [1] developed a method in which red blood cell membranes were solubilized by sodium dodecyl sulfate (SDS), and then the extracted membrane proteins were separated by acrylamide gel electrophoresis (SDS-PAGE). As a result, membrane proteins and glycoproteins could be detected on SDS gels. Figure 1 shows the typical human red blood cell membrane proteins separated by SDS-PAGE using the method of Laemmli [2], which was later improved by Fairbanks et al. [1]. Table 1 depicts the nomenclature of each cell membrane protein according to Fairbanks et al. [1], along with apparent molecular mass (kDa) and physiological function. Band 3, band 4.1, and band 4.2 are called the designated names at present.

**Figure 2:**
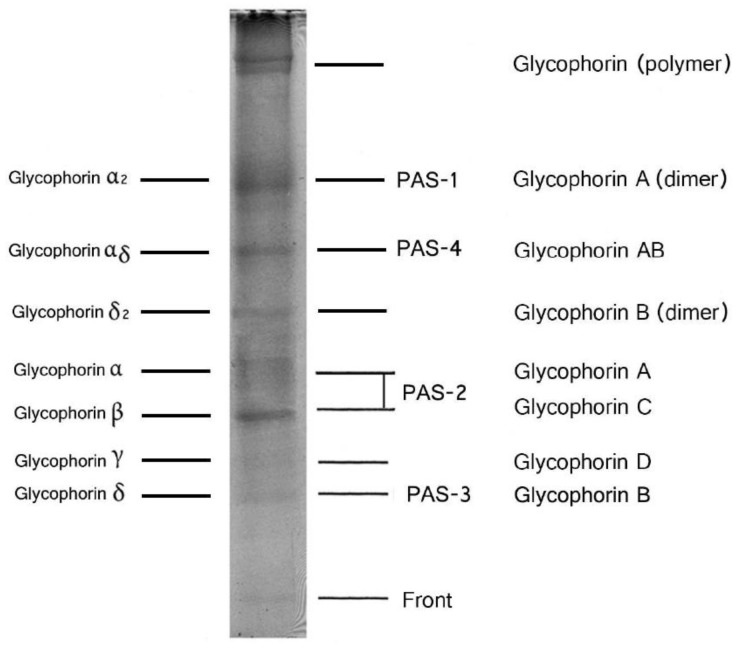
SDS-polyacrylamide gel electrophoresis of human red blood cell (RBC) membranes. Healthy human RBC membranes stained with periodic acid-Schiff (PAS) stain [1]. Electrophoresis was performed according to the method of Laemmli [2].

Band 3 is a glycoprotein and is detected as a diffuse band on SDS gel due to the microheterogeneity of the attached oligosaccharides [3]. Band 3 is detectable on the SDS gel by using protein staining with Coomassie Brilliant Blue (CBB) because band 3 has a small amount of oligosaccharides. Band 3 contains approximately 7% carbohydrate and contributes to approximately 10% of the total membrane carbohydrate [4]. By contrast, the sialooligosaccharide-rich glycoproteins are detectable by staining with the periodic acid-Schiff’s (PAS) reagent [1,5]. The glycoproteins detected by PAS staining are called glycophorins. Figure 2 shows the nomenclature of human red cell membrane sialoglycoproteins. The designation of glycophorins is confusing because different nomenclatures have been used by different researchers. In the first part of the 1970s, the glycophorins were called as PAS 1–4 and, at present, they are termed glycophorins A–D, respectively [6]. Glycophorin α is the same as glycophorin A, and glycophorin δ, β, and γ are glycophorins B, C, and D, respectively [6,7,8].

Glycophorin A (dimer) is observed below band 3 on SDS-polyacrylamide gels (Figure 2), is a major component of red cell membrane glycoproteins, and is reported to have an apparent molecular mass of 29 [9] to 36 kDa [10,11].

The electrophoretic migration of glycophorin is relatively low when compared to other membrane components because glycophorin is heavily glycosylated. Although the molecular mass of the other membrane proteins can estimated by migration on SDS-PAGE, the molecular mass of each glycophorin cannot be estimated in this manner.

## 2. Structure of the Human Red Blood Cell Membrane

**Figure 3:**
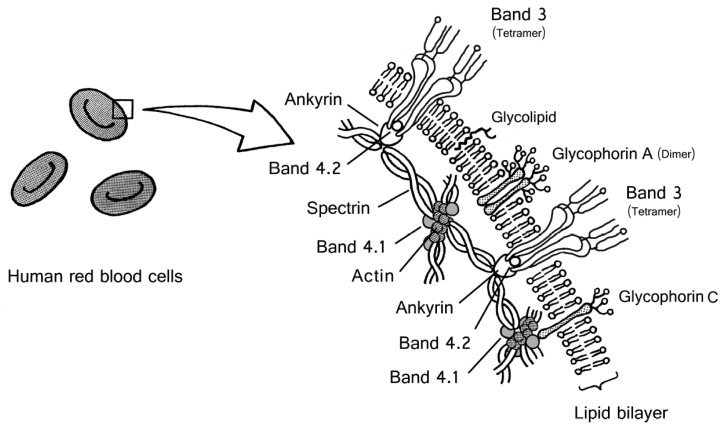
Illustration showing the location of band 3 and glycophorins in human red blood cell membranes. This illustration is based on several reviews [14,15,22]. According to Lux [14], the location of substoichiometric proteins (e.g., CD44, CD47, Lu, Kell, Duffy) remains unclear. In this illustration, these glycoproteins are omitted, the actin junctional complex (4.1R complex) is simplified, and the topology of band 3 and glycophorins is defined.

Red blood cell membrane proteins [12,13,14] can be classified into transmembrane proteins and peripheral membrane proteins that associate with the cytoplasmic side of the lipid bilayer. Band 3 and glycophorins are transmembrane proteins. Transmembrane proteins associate with the peripheral proteins that constitute the protein meshwork (cytoskeleton). Figure 3 shows a schematic drawing of the site of band 3 and glycophorins in human red blood cell membrane structure, with reference to the review on the red cell membrane skeleton [14,15]. The main component of the cytoskeleton is a spectrin tetramer. Its ends are linked by binding to the actin filament and band 4.1, and it forms a junctional complex. These junctions are linked to the end of some spectrin tetramers and form a netlike meshwork.

The N-terminal cytoplasmic domain of band 3 attaches to an intracellular protein (ankyrin), which binds to a spectrin tetramer. By connecting band 3 to ankyrin, band 3 links the cytoskeleton through the spectrin network. This cytoskeleton enables deformability and thereby maintains the integrity of the biconcave-shaped red blood cells.

Glycophorins C and D are linked to the band 4.1 protein and connect the cytoskeleton structure [16,17,18,19]. Glycophorins C, D, and band 3 are associated with the cytoskeleton, and the maintenance of shape and mechanical properties of the red blood cell after passing through capillary vessels [20].

This information indicates that glycophorins C and D are anchored to the membrane by the cytoskeleton. In contrast, there is only one report that glycophorin A connects to band 4.1 protein [21]. It is believed that glycophorins A and B are not associated with the cytoskeleton, thus enabling them to be easily released from the red cell membrane.

## 3. Structure of Band 3

The band 3 protein [3,23] is the most abundant of the red cell membrane proteins and composes approximately 25% of these proteins. For Band 3, approximately 1.0 × 10^6^ copies are present in each red blood cell. The molecular mass of band 3 is estimated at approximately 100 kDa by SDS-PAGE. By mild proteolysis, the band 3 protein, which consists of 911 amino acid residues, can be divided into a hydrophilic cytoplasmic fragment of 41 kDa and a hydrophobic membrane fragment of 52 kDa [24]. It forms a dimer or tetramer in the phospholipid bilayer, and this complex connects to the cytoskeleton through ankyrin.

The elongated part of the N-terminal domain facing the cytosol consists of a hydrophilic domain whose ends connect to the hydrophobic domain, and this unit crosses the membrane several times. The number of passes of the polypeptide unit has been established, and there may be 14 membrane spans based on cDNA analysis. The C-terminal domain is assumed to be on the cytoplasmic side of the membrane; however, the physiological function of this domain remains unclear.

**Figure 4:**
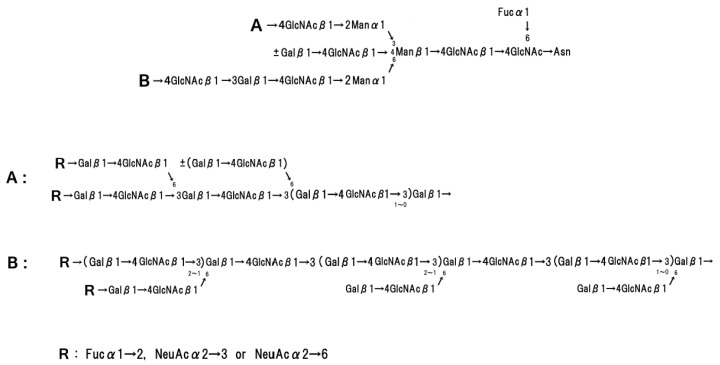
N-linked oligosaccharides of human band 3.

Recently, the crystal structure of the band 3 anion exchanger domain has been analysed in detail [25]. Band 3 is composed of amino peptides and a single N-linked oligosaccharide, and the molecular mass of the carbohydrate is approximately 8% of band 3. This N-linked oligosaccharide is linked at Asn-649, which is located at a site approximately 28 amino acids from the C-terminus of the band 3 polypeptide. This oligosaccharide is exposed on the external side of the membrane and is heterogeneous in size on different band 3 molecules, as determined by the number of repeating *N*-acetyllactosamine units (Galβ1→4 GlcNAcβ1→3) [26] (Figure 4). The end of the band 3 oligosaccharide is linked to sialic acid. This sialic acid is *N*-acetylneuraminic acid (5-acetamido-3,5-dideoxy-α-d-glycero-d-galacto-2-nonulopyranosidonic acid; Neu5Ac, NeuAc, NANA) and occurs broadly in humans [27].

## 4. Structure of Human Glycophorins

Glycophorins [10,28] A and B represent approximately 85% and 10% of the PAS-positive membrane components, respectively, whereas, glycophorins C and D are minor species, contributing only 4% and 1% to the PAS-positive components, respectively [29].

For the major sialo-glycoprotein, glycophorin A, it was deduced that 5 × 10^5^–10 × 10^5^ copies are present in each red blood cell [30], which corresponds to 1.6% of the total human red blood cell proteins [31]. By contrast, 0.2 × 10^5^–1.0 × 10^5^ copies of glycophorin B are present [32]. Glycophorin A consists of 131 amino acid residues as a single polypeptide chain [33] and contains 16 oligosaccharides attached to the N-terminus, accounting for a third of the molecule [34].

**Figure 5:**
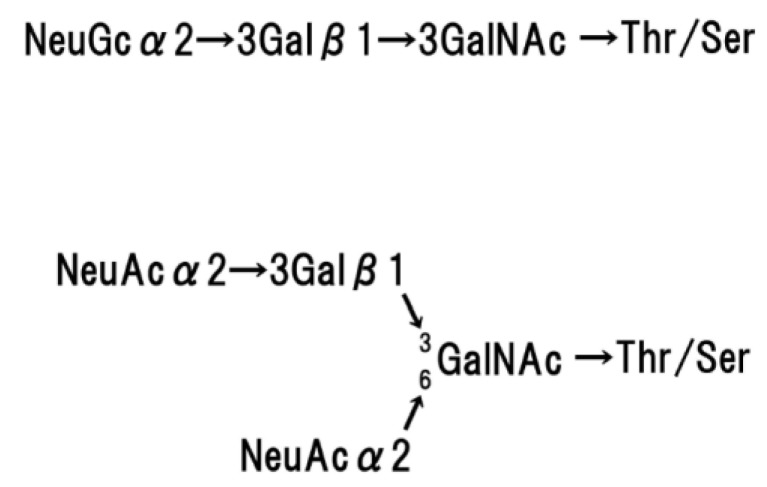
Basic structure of O-linked oligosaccharides of glycophorin A.

**Figure 6:**
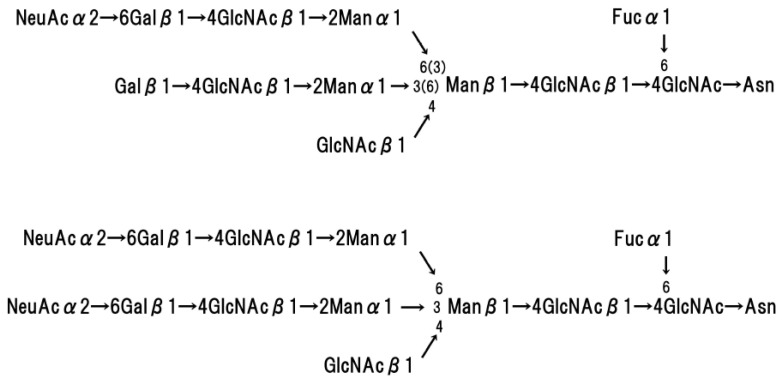
Basic structure of N-linked oligosaccharides of glycophorin A.

Glycophorin A homodimerizes in the red cell membrane [35,36,37], and this molecular structure has been analysed using NMR [38,39], polarized FTIR [40], and FRET measurements [41]. Glycophorin A is comprised of two motifs, an N-terminal moiety on the extracellular surface and a tethered C-terminal moiety, which consists of an α-helix (hydrophobic transmembrane domain) and a hydrophilic cytoplasmic domain. Glycophorin A is surrounded by approximately 34 phospholipids [42]. Fifteen O-linked oligosaccharide chains and a single N-linked oligosaccharide chain are attached to the extracellular N-terminal moiety. Figure 5 and Figure 6 show the basic structure of each oligosaccharide. The basic structure of the O-linked oligosaccharide consists of two NeuAc, Gal, and GalNAc, which form a tetra-saccharide, and the terminal GalNAc residue is attached to The/Ser of the polypeptide chain [43,44,45]. The basic structure of the N-linked oligosaccharide consists of NeuGc, Gal, Man, Fuc, and GlcNAc, and the terminal GlcNAc residue is attached to Asp 26 of the glycophorin polypeptide [46]. The amount of sialic acid contained in glycophorin A corresponds to approximately 75% of the total sialic acid of the red blood cell membrane [47].

Glycophorin B was isolated by Furthmayr et al. [31,48] using the lithium diiodosalicynate (LIS)-phenol method [49]. The molecular mass of glycophorin B is estimated at approximately 20 kDa, and the sequence of N-terminal domain is identical to that of glycophorin A [11,50] because both of the glycophorins are generated from the same gene cluster [51,52,53]. Glycophorins C and D are also generated from the same glycophorin gene cluster, and their molecular weights are estimated at approximately 32 kDa and approximately 23 kDa, respectively [54,55]. Glycophorins C and D have been deduced to occur at approximately 5.0 × 10^4^–10 × 10^4^ and approximately 2.0 × 10^4^ copies in each red blood cell, respectively [56].

Glycophorin B has no N-linked oligosaccharide and 11 O-linked oligosaccharides bound to the protein moiety; glycophorin C has a single N-linked oligosaccharide and 12 O-linked oligosaccharide chains; and, glycophorin D contains six O-linked chains [56,57]. The structures of the oligosaccharides of the other glycophorins are similar to those of glycophorin A [48]. In 1990, a gene for a fifth glycophorin (glycophorin E) was identified by the isolation of genomic clones, and its nucleotide sequence is almost identical to that of the glycophorin B gene [58,59]. However, the expression and the function of glycophorin E have not been clarified [60].

## 5. Other Glycoprotein in the Human Red Blood Cell Membrane

Although they have not been detected by PAS staining of SDS-PAGE gels, some glycoproteins in human red blood cell membranes have been identified by using monoclonal antibodies.

There are reports that a band 4.5 protein (GLUT1) contains N-glycan, and increasing the *N*-glycosylation of GLUT1 may influence its function as a glucose transporter [61].

The Na-H exchanger (NHE1), which associates with actin-binding proteins (ERM: ezrin, radixin, and moesin), contains both N-linked and O-linked oligosaccharides [62]. Its isoform, NHE2, has only O-linked oligosaccharides [63]. However, glycosylation of NHFs had no apparent effect on the rate of ion exchange [64]. CD44 and CD47 are also glycoproteins. The former binds to protein band 4.1 (4.1R), and the latter binds to band 4.2 (protein 4.2) and to the ankyrin-linked band 3 complex [14]. In non-erythroid cells, CD44 binds directly to ERM proteins [65]. CD44, which is involved in cell-cell communication, is known to be the cell surface receptor for hyaluronan (HA) and contains *N*-glycans, *O*-glycans, and glycosaminoglycans [66]. It has been reported that the glycosylation of CD44 affects its HA affinity [67,68]. CD47, which is also involved in cell-cell communication, carries *N*-glycans [69] but does not require glycans to interact with signal regulatory protein α (SIRPα) [70]. The physiological function of these glycans seems to be providing structural integrity, similar to LPS attached to the outer cell membranes of gram-negative bacteria.

In addition to glycophorins, several glycoproteins in human red cell membranes have blood group antigens [71]. DARC/Duffy blood group antigens have been reported in proteins that contain N-glycan [72] and in the receptor for the malaria parasite [73]. Lu (Lutheran) blood antigen, which enables binding to α-spectrin, appears to contain small amounts of both N-glycan and O-glycan [74]. These glycans are affected by the expression of Lu^b^ antigenic activity [75]. Although present in only small amounts, the Kell blood group is a major antigenic system in human RBCs, and it interacts with band 3 protein [76]. This antigen is carried by a 93 kDa glycoprotein, and it has six N-linked polysaccharides that do not contain NeuAc [77,78]. However, the physiological function of Kell antigen is related to its amino acid composition, not polysaccharides [79], in contrast to I blood type antigen (the poly-*N*-acetyllactosamine structure) [80]. The diversity of blood group antigens, as mentioned above, which is lacking in carp sera (in the Section 9), may be related to the existence of various anti-carbohydrate antibodies in human sera [81]. Researchers are interested in the reactions of these antibodies with oligosaccharides containing mainly NeuAc, Gal, and Fuc at the termini of polysaccharides, but these antibodies do not react with Man, which is the primary component of N-glycan.

## 6. Physiological Function of Band 3

Band 3 [3,82,83,84] is well known to function as an anion exchanger. When red blood cells pass through the lung blood vessels, band 3 protein has a function in collecting CO_2_ from human tissues in exchange for Cl^−^ as the form of HCO_3_^−^. Moreover, such an anion exchange function is also involved in pH regulation within cells. There are several reports on the band 3-like membrane protein having these functions in various cell membranes [84,85].

The role of the N-linked oligosaccharide of band 3 has not been established. There is a report that the oligosaccharide of band 3 does not affect the anion transport function in red blood cells [86]. Other reports indicate a role for the oligosaccharide as an antigen, and these reactions are related to the ageing phenomenon [87,88,89]. In this reaction, when the red cell membrane is damaged by oxidation through the ageing process, the localization of band 3 changes to bind anti-band 3 IgG (senescent antigen), and this binding site is at the oligosaccharide that has terminal NeuAc residue. The anti-band 3 IgG that binds band 3 is recognized in the spleen by macrophages, and macrophage autolysis digests the senescent red blood cell [90,91,92,93]. However, other reports demonstrate that the anti-band 3 IgG was bound to the band 3 protein itself, not the carbohydrate moieties [94,95].

## 7. Physiological Function of Human Glycophorins

Glycophorins [30,96] are extracted from red cell membrane preparations in the aqueous phase using organic solvents with some detergents because of their high content of sialic acid and highly hydrophobic protein moiety. Several organic solvents have been used as extract solutions, mainly chloroform/methanol [97], pyridine [98], and LIS-phenol [49]. The obtained glycophorin preparation is highly prone to aggregate, even in the presence of SDS [99,100,101,102]. Glycophorin A (PAS 1) forms a dimer in the SDS gel in SDS-PAGE [100,103,104]. Moreover, it forms a polymer [50] or hybridizes with glycophorin B (PAS-4, α-δ-glycophorin) [105,106]. This dimerization is caused by the α-helix of the transmembrane domain of glycophorin A interacting with another glycophorin in the intact red cell membrane [37,40]. Glycophorin B also forms a dimer, similar to glycophorin A [18].

As mentioned above (in Section 2), glycophorins C and D are associated with the cytoskeleton and the maintenance of the shape and mechanical properties of red blood cells [21], and this function contributes to the negative surface charge caused by glycophorins containing sialic acid. Without its negative charge, the mobility of the red blood cell is greatly reduced [107].

The major glycophorin, glycophorin A, is a single polypeptide chain and is linked to several sialo-oligosaccharides, which carry some blood group antigens. These blood group antigens are located at the O-linked oligosaccharides and the nearby amino acid sequence of these oligosaccharides binding site. Glycophorin A carries the M and N blood group antigens, as determined by the amino acid sequence at residue 1 and residue 5 of the mature polypeptide (Ser1/Gly5 for M and Leu1/Glu5 for N). Glycophorin B, on the other hand, has only an N form, and its amino acid sequence is identical to that of N-type glycophorin A. Glycophorin B carries other blood group antigens, the Ss blood group, as determined by the amino acid sequences at Met 29 for S or Thr 29 for s specificity [108].

Both glycophorin polypeptides possess 3 O-linked oligosaccharides containing NeuAc linked at amino acid residues 2, 3, and 4. Both glycophorins lose blood group activities by desialylation treatment [109]. The Wright (Wr) antigens are also reported to be associated with band 3 and glycophorin A [110]. Glycophorin C and its shorter form, glycophorin D, are antigenically distinct from glycophorins A and B. Glycophorin C carries several blood group antigens (Gerbich, Yus, Wb, An^a^, and Dh^a^, and others) [111,112,113]. Glycophorins are also reported to carry the ABH blood group antigens [114,115]. According to Podbielska et al. [116], O-linked oligosaccharides isolated from glycophorin A carried the A, B, or H blood group antigen. Although these oligosaccharides reacted with ABH blood group antigens, the reaction was estimated at a relatively low level. It is thought that the oligosaccharides containing Fuc are minor components in the total amount of oligosaccharides from glycophorin A.

Another physiological function of glycophorins involves the lectin receptors. Glycophorins are linked to several sialo-oligosaccharides, so several lectins, such as *Psathyrella velutina* lectin (PVL), wheat germ agglutinin (WGA), and others, interact with the oligosaccharide moiety of glycophorin [117,118,119].

Other functions of glycophorins as receptors are reported to be involved with the vital functions of interaction of red blood cells and the influenza virus [120,121], Sendai virus [122,123], malaria parasite [124,125,126,127], and *Escherichia coli* α-haemolysin [128]. Although the relation between influenza virus and human red blood cells was known as early as the 1940s [129], there was difficulty proving this reaction because glycophorin is prone to aggregation, and the use of organic solvents and contaminating glycolipids in the purification process need to be considered. In the 1990s, the vital function became clear after the development of new experimental methods (e.g., using detergents [130], reconstruction of glycophorin-bearing liposome by using egg phosphatidylcholine [120], quick-freezing electron microscopy [121], and elastic light scattering [131]). Recently, experiments performed with stem cell lines revealed the interaction of glycophorin A with band 3 [132], and glycophorin C as a receptor for the rodent malaria parasite [133].

Several lines of evidence have suggested an interaction between glycophorin A and band 3 in their biosynthesis and processing [3,134]. However, glycophorin A knockdown had no effect on the cell surface expression of band 3 [110]. Moreover, glycophorin A-deficient red blood cells did not clearly demonstrate the physiological role of glycophorin A [135].

## 8. Glycoproteins in Red Cell Membranes of Non-Human Origin

Although glycoproteins in the human red cell membrane have been researched extensively, there are few reports on glycoproteins in other mammalian or avian red cell membranes. Some researchers have reported the electrophoretic patterns of mammalian red cell membranes (ox, horse, swine, sheep, goat, rabbit, guinea pig, rat, and mouse) [136,137]. According to these reports, these main membrane proteins are similar to those of human red cell membranes. Each membrane component (ankyrin, spectrin, band 3, band 4.1, and actin) was detected using SDS gels. The electrophoretic pattern of the cat red blood cell membrane (*Felis catus*) was also similar to that of human [138].

**Figure 7:**
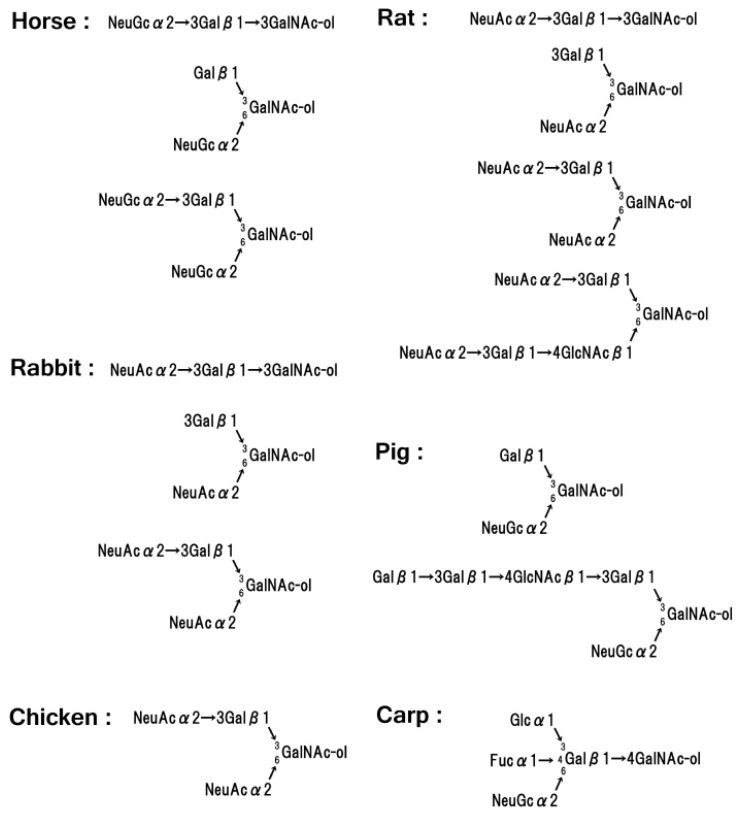
O-linked sialo-oligosaccharides of glycoproteins of mammalian, avian and teleost origins.

On the other hand, glycophorin patterns (PAS-stained band patterns) are different in humans [136], and these differences are caused by the component containing oligosaccharides. The glycophorins from horse [139], bovine [140], pig [141], and goat contain sialic acid as NeuGc (*N*-glycolylneuraminic acid), not as NeuAc [142]. While the sialic acid of dog [143] and mouse [144] is NeuAc, canine individuals containing only NeuGc have been reported. The monkey glycophorin demonstrates MN blood group activities and contains a single O-linked oligosaccharide that is composed of NeuGc, Gal, and GalNAc [145]. The core structural unit of these O-linked sialo-oligosaccharides from various mammalian sources is sialic acidα2→3Galβ1→3(sialic acidα2→6)GalNAc-ol or sialic acid α2→[3Galβ1→4GlcNacβ1]_*n*_→3Galβ1→3GalNAc-ol [146] (Figure 7).

Non-mammalian red blood cell membranes have not been researched extensively. A major reason for this is that avian and teleost red blood cells contain a nucleus, and this causes difficulty during cell membrane preparation. The red blood cell membranes of pigeon [147], chicken [148], and turkey [149] for avian species and rainbow trout [150] for teleosts have been reported, and the main membrane proteins are similar to those of human red blood cells. Research on chicken band 3 [151] and rainbow trout band 3 [152,153] has been reported, while detection of glycophorins (PAS-stained bands on SDS-gel) has been reported based on chicken membrane preparation, and the core structure of the O-linked oligosaccharide is the tetraose NeuAcα2→3Galβ1→3(NeuAcα2→6)GalNAc-ol [154,155] (Figure 7).

There were no reports on teleost glycophorin until Aoki et al. reported the detection of glycophorins in carp and rainbow trout red cell membranes [156].

## 9. Glycophorin in Carp Red Blood Cell Membranes

Aoki et al. reported the presence of glycophorins in red blood cell membranes of carp on SDS electrophoresis gels by PAS staining [156]. While membrane proteins from carp membrane preparations are similar to those of human red cell membranes, carp and trout showed different glycophorin patterns. The major glycophorin from carp membrane preparations is positioned near the human glycophorin A (dimer). According to the amino acid composition of carp glycophorin, there was no striking difference from that of human glycophorin A [157]. Although human glycophorins A and B carry the MN and Ss blood group antigens, it is unclear whether carp glycophorin carries these blood group antigens, as no blood group antigen reaction has been observed by titration (Aoki et al., unpublished materials).

The O-linked oligosaccharide of carp glycophorin exhibited bacteriostatic activity, and this activity is observed on all tested bacteria (Gram-positive bacteria: *Micrococcus luteus* and *Bacillus subtilis*, Gram-negative bacteria: *Vibrio anguillarum*, *Edwardsiella tarda*, *Aeromonas hydrophila, Escherichia coli*, and *Pseudomonas fluorescens*) [157,158]. In the blood of diseased carp infected by *P. fluorescens*, carp glycophorin is released from red blood cell membranes and interacts with the bacterium [158]. By electron microscopic observations, the released carp glycophorin molecule from the cell membrane attaches to the flagellum of *V. anguillarum* or the cell surface of *M. luteus* and inhibits bacterial growth [157].

**Figure 8:**
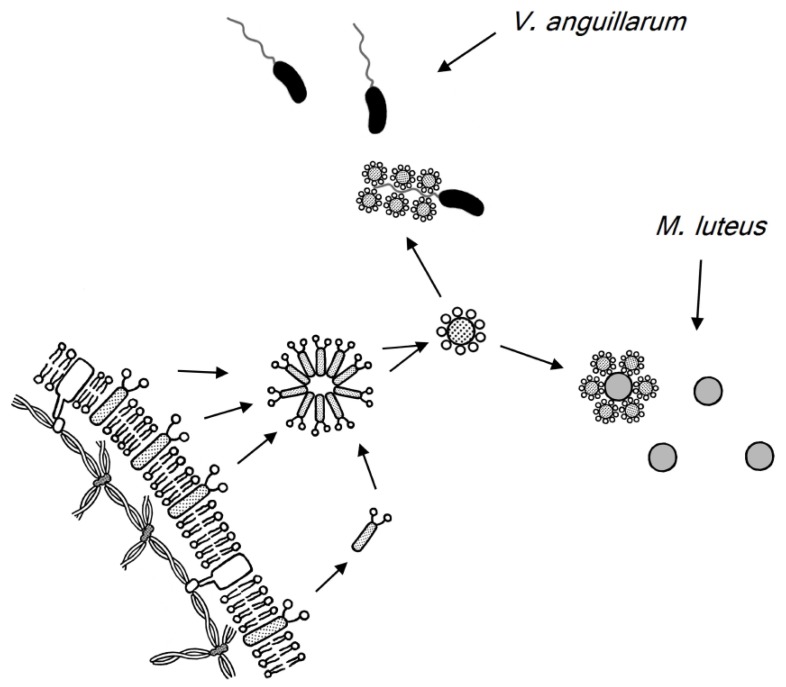
Schematic representation of the carp glycophorin interaction with invading bacteria in carp blood.

These bacteriostatic activities are caused by the sialo-oligosaccharide from carp glycophorin and are attributed to the nature of the lectin receptor. It is thought that some lectin-like proteins exist on the surface of Gram-positive bacteria or the flagellum of Gram-negative bacteria. These observations indicate that carp glycophorin is released from red cell membranes and adsorbed onto the surface of invading bacteria in the blood (Figure 8).

In the Edo period of Japan, drinking carp blood was well known to the people as a folk remedy for tuberculosis. As tuberculous is caused by the bacterium (*Mycobacterium tuberculosis*), the bacteriostatic activity of carp glycophorin is suggested to relate to the efficacy of drinking carp blood.

The structure of the bacteriostatic sialo-oligosaccharide of carp glycophorin was determined as NeuGcα2→6(Fucα1→4)(Glcα1→3)Galβ1→4GalNAc-ol [159] (Figure 7). The 1→4 linkage of GalNAc is unique as compared with other O-linked oligosaccharides of mammalian origin. Interestingly, the β1→3 glycosidic linkage of xylan, which is a component of seaweed cell walls, is unlike the standard β1→4 linkage of land plants [160]. It is possible to detect the β1→4 linkage of *N*-acetylgalactosamine in marine organisms.

The sialo-oligosaccharides from carp glycophorin that have bacteriostatic activity are pentoses. This may be related to the finding that penta- or hexasaccharides obtained from chitin have bacteriostatic activity [161]. In the bacteriostatic reaction, it is supposed that the size of the oligosaccharide would correspond to the dimension of the cleft that occurs in the lectin-like protein, and might contribute to the charge of sialic acid. In teleost blood, IgG does not exist, and other antibodies exist in low levels [162]. It is suggested that glycophorin may exist as a substitute for antibodies in teleost blood. Although the physiological function of human glycophorin has not yet been clarified, the structure of the human glycophorin O-linked tetra oligosaccharide is a simpler form than that of the carp’s pentose. It is considered that IgG became a major component in the human immune system and that the bacteriostatic activity of human glycophorins has been lost in the process of evolution.

## 10. Conclusions

The research on glycosylation of RBC membrane proteins has not been fully investigated when compared to the research on the protein moiety of RBC glycoproteins. Moreover, the target of research on RBC is mainly human blood. Although the preparation of human RBC membranes is relatively easy, the component of human RBC is more complicated than non-mammalian RBC, especially in regard to the blood group antigens.

On the contrary, the preparation of teleostei RBC membranes is fairly difficult because containing cell nucleus, the component of RBC is simpler than that of human. In particular, the glycosylation of carp RBC is simpler than that of human glycophorins A, B, C, and D. As mentioned above, accordingly, it is suggested that the collection of information on the non-mammalian RBCs leads the comprehension on the role of human RBC glycosylation.

## Figures and Tables

**Table 1:** Red blood cell membrane proteins.

Band	Molecular Mass (kDa)	Designation	Function
1	240	spectrin (α chain)	components of cytoskeleton
2	220	spectrin (β chain)	
2.1	210	ankylin	
3	100	band 3 (AE1)	anion transporter
4.1	82	band 4.1	components of cytoskeleton
4.2	76	band 4.2	
4.5	55	band 4.5 (GLUT1)	glucose transporter
5	43	actin	components of cytoskeleton
6	35	glyceraldehyde-3-phoshate dehydrogenase (GAPDH)	glycolytic enzyme
7	31	stomatin	–
